# HIV associated cell death: Peptide-induced apoptosis restricts viral transmission

**DOI:** 10.3389/fimmu.2023.1096759

**Published:** 2023-02-22

**Authors:** Qiongyu Chen, Yan Zhao, Yonghong Zhang, Jianbo Zhang, Wenshu Lu, Chih-Hao Chang, Shisong Jiang

**Affiliations:** ^1^ Department of Oncology, University of Oxford, Oxford, United Kingdom; ^2^ The Jackson Laboratory, Bar Harbor, ME, United States; ^3^ You’an Hospital, Beijing, China; ^4^ The Dermatology & STD Department, The No. 2 People’s Hospital of Dali City, Yunnan, China; ^5^ R&D Department, Oxford Vacmedix (Changzhou) Ltd, Changzhou, Jiangsu, China

**Keywords:** HIV, apoptosis, necrosis, transmission, T-cells, peptides

## Abstract

The human immunodeficiency virus (HIV) is still a global pandemic and despite the successful use of anti-retroviral therapy, a well-established cure remains to be identified. Viral modulation of cell death has a significant role in HIV pathogenesis. Here we sought to understand the major mechanisms of HIV-induced death of lymphocytes and the effects on viral transmission. Flow cytometry analysis of lymphocytes from five latent HIV-infected patients, and HIV IIIB-infected MT2 cells demonstrated both necrosis and apoptosis to be the major mechanisms of cell death in CD4^+^ and CD4^-^/CD8^-^ lymphocytes. Significantly, pro-apoptotic tumor necrosis factor (TNF) peptide (P13) was found to inhibit HIV-related cell death and reduced viral transmission. Whereas pro-necrotic TNF peptide (P16) had little effect on HIV-related cell death and viral transmission. Understanding mechanisms by which cell death can be manipulated may provide additional drug targets to reduce the loss of CD4^+^ cells and the formation of a viral reservoir in HIV infection.

## Introduction

After 40 years, the AIDS pandemic is by no means ending. Yet the Covid pandemic has redirected some AIDS-related resources to the new disaster which has compromised AIDS prevention and management (1). In 2020, the human immunodeficiency virus (HIV) led to almost one and a half million deaths globally, with 680,000 new infected with HIV (2). HIV infection leads to a decline in CD4^+^ T cell counts and the production of viral reservoirs (3, 4). Major advances in the treatment of HIV infection have improved overall survival rates and reduced progression to acquired immune deficiency syndrome (AIDS) (1, 4, 5). However, patients living with HIV under the control of highly active antiretroviral therapy (HAART) are at risk of disease-associated infections, chronic inflammation, and immune-senescence (6).

HIV infects CD4^+^ cells *via* the gp120 envelope protein interacting with the CD4^+^ receptor and co-receptors CCR5 or CXCR4, by M- and T-tropic strains respectively (7). Following reverse transcription to form proviral DNA in the cytoplasm, the virus integrates into the host genome where it can replicate and produce a pool of infected cells (8, 9). For reproduction purposes, HIV has to evade killing by host immunity and spread the virus from one host cell to another. The virus engages several strategies to reach its goal. For example, HIV is an RNA virus with highly error-prone reverse transcriptase that leads to viral mutations and evolution. When the proviral DNA does not integrate into the host genome by perhaps an epigenetic mechanism, the virus becomes dormant and stays in the host cell for a long time without being killed. This is called latency which is another strategy that HIV uses to avoid being destroyed.

HIV also aims to disturb the immune system to benefit its reproduction and spreading strategy. It infects CD4^+^ T cells which are key for coordinating cellular and humoral immune responses. Depletion of CD4^+^ T cells will weaken both CD8^+^ T cells which are important to clear viruses and B cells which produce neutralizing antibodies to prevent cell free viruses from entering cells. Consequently, the virus will replicate and spread to infect other cells. There are several HIV-related CD4^+^ T cell deaths namely apoptosis, necrosis, and pyroptosis (10). HIV viral proteins have been well characterized for their pro-and anti-apoptotic functions (11). HIV viral proteins can lead to survival or apoptosis of infected cells, and apoptosis of uninfected bystander CD4^+^ T cells, contributing to the formation of a viral reservoir and immunodeficiency (11–13).

Apoptosis is a type of programmed cell death whereby cells initiate their own demise in response to a stimulus, which has been a well-studied mechanism of cell death in HIV infection (3, 4). Caspase-3 is known as an executioner caspase that plays a key role in the apoptotic process. However, other types of cell death have been found to be involved in the pathogenesis of HIV. Necrosis has previously been described in human CD4^+^ cells infected with HIV according to morphological and DNA fragmentation patterns (14). Necrosis is a premature cell death characterized by swelling of cytoplasmic compartments and the absence of caspase-3 signaling; therefore, it can be detected by measuring the permeability of the plasma membrane to a normally impermeable fluorescent dye, such as a live-dead DNA-binding dye. Pyroptosis *via* caspase-1 signaling was also found to have major involvement in the loss of non-productively infected CD4^+^ cells in HIV infection of human lymphoid tissue (15). Other cell death pathways with characterized roles in HIV include autophagy and activation-induced cell death (16, 17). Therapies are being developed to target apoptotic pathways (11). Despite this, a treatment that targets CD4^+^ T cell death (including bystander-killing) in HIV infection remains to be established. Further understanding of HIV-mediated cell death may lead to the development of further therapeutic targets.

Targeting cell death as a treatment for HIV is an attractive idea (18, 19). However different cell deaths induced may lead to different consequences. For example, apoptosis is a programmed cell death with shrinkage of the cells, intact cell membrane, and condensed and fragmented DNA/chromatin. Apoptosis of HIV-infected cells may contain the viruses inside the dead cells without viral spreading to further infect other cells. On the other hand, necrosis is a premature cell death with a disrupted cell membrane and the release of cellular contents (such as uric acids and genetic materials) to the extracellular milieu. For necrosis of HIV-infected cells, the viruses may be released and transmitted to other target cells. The benefit of necrosis may be that it may facilitate the “shock and kill” strategy to treat the latency of HIV infection. To develop an optimal therapeutic method of cell death, it is necessary to understand the effect of different cell death on HIV-infected cells. This will be helpful for choosing different strategies for achieving different goals of treatment.

The aim of this study is to identify cell death patterns in patients with latent HIV infection (anti-HIV antigen positive without any HIV-related symptoms) and to understand the consequences of apoptosis and necrosis on HIV-infected cells. The key reagents used in this project are two peptides derived from tumor necrosis factor (TNF). TNF binds to the tumor necrosis factor receptors (TNFR) and causes pro-inflammatory signaling or cell death (20). TNF may induce apoptosis or necrosis under certain conditions. We have previously identified two conserved peptide regions of TNF, one which is responsible for inducing apoptosis (P13) and the other for necrosis (P16) (21). The two peptides are derived from the same molecule and yet carry out pleiotropic functions. To further understand the effect of apoptosis and necrosis on HIV-mediated pathogenesis and transmission, we use the two peptides identified to investigate the effect of different cell deaths on the viral load of HIV-infected cells.

## Materials and methods

### Study design

The goal of this study was to understand the type of cell death in latent HIV infection and the effects of two different cell deaths, apoptosis or necrosis, on viral transmission. We first recruited five patients newly diagnosed with HIV infection through an HIV clinic in China. The cohort was tested for HIV at regular screenings and diagnosed using western blots. These patients did not have any HIV-related symptoms when they were recruited in this study. Healthy volunteer adults between the ages of 20 and 45 were eligible for enrollment. We conducted the studies in compliance with Good Clinical Practice guidelines (CPMP/ICH/135/95) and the Declaration of Helsinki. They were all studied with informed consent in full compliance with national and institutional ethical requirements (ethics approval number D0906003000091). All samples were stored at −80°C until analysis.

### Cell lines, TNF, TNF peptides, and HIV

MT2 cells were purchased from American Type Culture Collection (ATCC, Manassas, Virginia, US) and maintained in RPMI supplemented with 10% fetal calf serum and penicillin/streptomycin (complete medium). All cultures were incubated at 37°C in 5% CO_2_. Human TNF was purchased from Immunotools (Friesoythe, Germany). Peptides were synthesized on an automatic APEX 396 using a standard solid phase Fmoc strategy. In some of the validation experiments, peptides (e.g. P13 and P16 peptides) were purchased from Proimmune (Oxford, UK). HIV-1 strain IIIB (1000 TCID50) kindly provided by Professor Tao Dong (University of Oxford, UK) was used to infect cells *in vitro*.

### Apoptosis and necrosis assay

HIV-1 IIIB virus at a multiplicity of infection of 0.001 was adsorbed to MT2 cells in serum-free RPMI, 1 mM L-glutamine, 0.1% bovine serum albumin for 1 hour at 37°C. Cells were then supplemented with the complete medium in the presence or absence of DMSO-dissolved whole TNF protein or TNF-derived peptides and incubated for additional 24 hours at 37°C. The cells were stained with live/dead cell-staining kits (Invitrogen, Paisley, UK) according to the manufacturer’s instructions and fixed using cytofix/cytoperm fixation/permeabilisation solution kits (BD Pharmingen, Oxford, UK). This was followed by intracellular staining with the FITC-conjugated anti-caspase-3 antibody (Cell Signaling Technology, Danvers, MA, USA). HIV-1 Gag p24 protein was detected by PE-conjugated anti-p24 monoclonal antibody (Beckman Coulter, Brea, CA, USA). All other flow cytometry antibodies were purchased from BD Biosceinces. Caspase inhibitor z-VAD (R&D Systems, Lake Bluff, IL, USA) was dissolved in DMSO and used at 10 µM in this study. Cells were acquired on a CyAn flow cytometer (Beckman Coulter, Fullerton, CA, USA) and data were analyzed using Flowjo (Tree Star Inc. Ashland, Oregon, USA).

### Statistical analysis

Comparisons for two groups were calculated by multiple, unpaired two-tailed t-tests with Prism 9 (GraphPad Software, San Diego, CA, USA). Statistical significance was defined at the following levels: * *p* < 0.05, ** *p* < 0.01, *** *p* < 0.001, and ns (not significant, *p* > 0.05).

## Results

### In HIV infected patients: Latent HIV infection results in apoptosis and necrosis of CD4^+^ CD8^-^ and double negative lymphocytes

**Figure 1:**
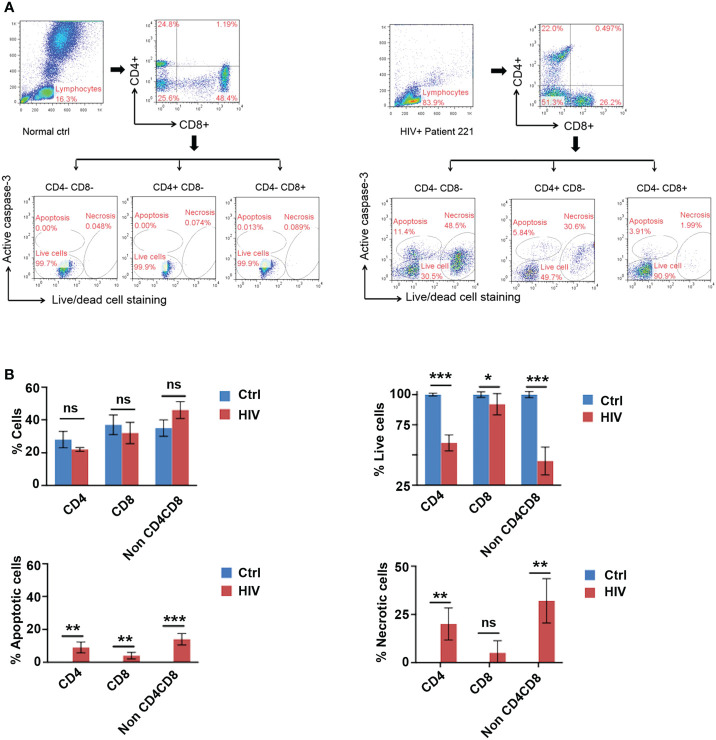
Identification of Apoptosis and Necrosis in Lymphocytes of HIV Infected Patients. **(A)** Gating strategy: Peripheral blood lymphocytes were first gated in the FSC/SSC (forward scatter/side scatter) properties. Within this population, CD4^+^ T cells and CD8^+^ T cells were then identified based on their specific surface markers. A live-dead fixable stain in combination with an active caspase-3 antibody was used to differentiate between apoptosis (Caspase-3^+^ live-dead^−^), necrosis (Caspase-3^−^ live-dead^+^), and live (Caspase-3^−^ live-dead^-^) cells. Representative flow cytometry plot for cell death of lymphocytes in an HIV-infected patient (#221, right) and a healthy individual 1 (#1, left). **(B)** HIV infection led to cell death by apoptosis and necrosis in CD4^-^/CD8^-^, and CD4^+^ lymphocytes. Frequencies of live lymphocytes and their apoptotic/necrotic death in HIV infected patient blood (HIV) as measured in **(A)** in comparison with those in healthy individuals (ctrl). Data shown as mean ± SD. *p < 0.05, **p < 0.01, ***p < 0.001, and ns, *p* > 0.05 (Multiple unpaired t-test).

HIV infects T cells *via* its receptor, CD4, together with its co-receptor, CCR5 or CXCR4, ultimately causing cell death (22, 23). To reveal whether and how T cells in HIV infected patients die as a result of HIV infection, we investigated the death of blood lymphocytes from five latent phase patients (**Table 1**). Lymphocytes were gated according to their size (forward scatter/side scatter). Caspase-3 and live-dead dye double staining was used to detect apoptotic and necrotic cells *via* flow cytometry. A higher degree of cell death by apoptosis (Caspase-3^+^ live-dead^−^) and necrosis (Caspase-3^−^ live-dead^+^) was found in both CD4^+^ CD8^-^ T cells and double negative (CD4^-^, CD8^-^) lymphocytes of the patients than in CD4^-^ CD8^+^ T cells and those of the healthy individuals (**Figure 1A**). A summary of flow cytometry analysis of cell death in five patients shows a decrease in the percentage of live CD4^+^ and double negative lymphocytes in HIV infected individuals compared to five healthy controls (**Figure 1B**). Compared to healthy individuals, a higher proportion of peripheral lymphocytes in HIV patients were found to be undergoing cell death, with little to no cell death observed in healthy individuals. Additionally, the frequency of necrotic cells was found to be higher among HIV patients than that of apoptotic cells (**Figure 1A**, lower right panel; **Figure 1B** lower panels). These data suggest that latent HIV infection can promote a greater frequency of necrosis in patient lymphocytes, with a lesser degree of apoptosis observed.

**Table 1:** Brief clinical data.

ID	Age	Gender	Date sample collecting	Sexual orientation	MSM history	HIV Symptoms	HIV diagnosis*	CD4 count	CD8 count
Patient 211	41	M	02/08/2010	MSM	16 years	0	+	234	520
Patient 222	24	M	12/08/2010	MSM	3 years	0	+	665	1852
Patient 223	20	M	20/09/2010	NA	NA	0	+	550	2048
Patient 224	25	M	26/07/2010	MSM	2 years	0	+	599	1908
Patient 225	21	M	27/07/2010	MSM	4 months	Fever+ Diarrhoea	+	637	2219
Healthy 1	25	M	20/08/2010	F	–	0	–	750	722
Healthy 2	30	M	26/08/2010	NA	–	0	–	993	501
Healthy 3	33	M	06/09/2010	F	–	0	–	860	864
Healthy 4	44	M	15/09/2010	F	–	0	–	722	550
Healthy 5	22	M	29/09/2010	F	–	0	–	1149	635

*Western blot tests positive for serum antibodies against HIV protein (p160, p120, p41, and p24). M, male; F, female; MSM, men sex with men; NA, not available.

### *In vitro* HIV infection model: Apoptosis and necrosis induced by HIV

**Figure 2:**
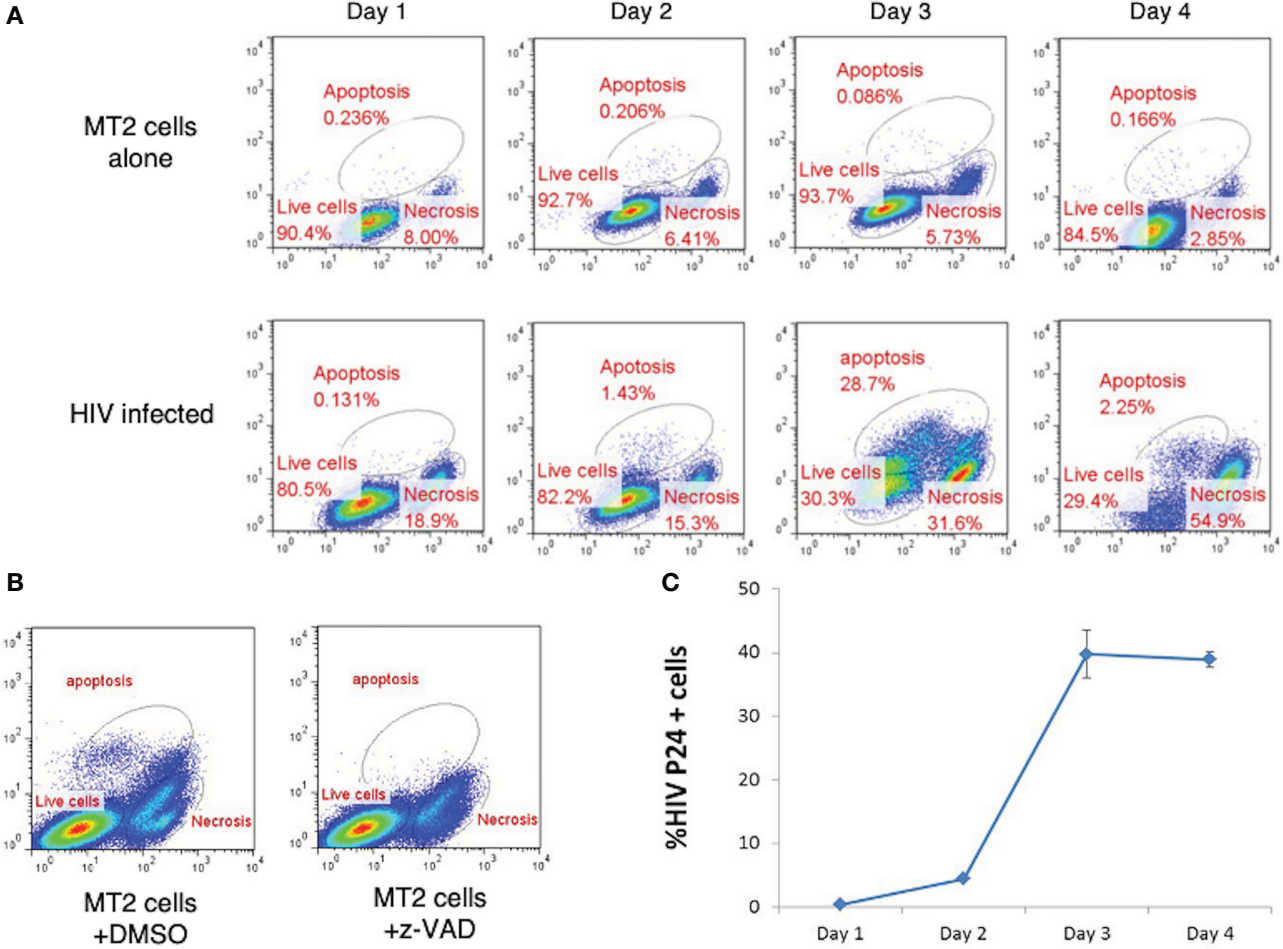
Effect of HIV Infection on Cell Death in MT2 Cells. **(A)** HIV infection led to cell death by apoptosis and necrosis. MT2 cells were infected at day 0 with HIV IIIB (MOI 0.001). Cell death was determined by live-dead dye (X-axis) vs. Caspase-3 (Y-axis) double staining and measured over four days by flow cytometry. **(B)** Flow cytometry dot plots show apoptosis of MT2 cells was inhibited with the presence of z-VAD (10 µM) left overnight but not DMSO. **(C)**. HIV p24 detected by PE-labeled anti-p24 antibody was used to measure the percentage of MT2 cells infected with HIV IIIB (MOI 0.001) over 4 days. Data shown as mean ± SD. These data are representative of three independent experiments.

To study HIV related lymphocyte cell death, it is necessary to set up an *in vitro* model. In the presence of HIV, infected cells died by apoptosis and necrosis, which can be detected with flow cytometry (**Figure 1**). We therefore investigated the dynamics of HIV-induced lymphocyte death by infection of an MT2 CD4^+^ T cell line with HIV strain IIIB. Our data revealed that MT2 cells maintained relatively stable survival rate of approximately 85-94% over the 4-day culture period, with a small proportion of cells, approximately 0.09-0.23%, undergoing apoptosis, and 2.9-8% undergoing necrosis (**Figure 2A** upper). Notably, in the HIV-infected cells, the rate of apoptosis peaked at day three (28.7%), whereas the rate of necrosis peaked at day four (54.9%) (**Figure 2A** lower). Specific caspase inhibitor z-VAD was used to confirm that the gated apoptosis area is correct (**Figure 2B**). The percentage of cells with positive HIV p24 (used as a measure of viral load) was measured by flow cytometry and found to increase rapidly over the course of the four-day infection (**Figure 2C**). The established *in vitro* HIV infection model is suitable to screen peptides with therapeutic potentials.

### Apoptosis-inducing TNF peptide reduces viral load

**Figure 3:**
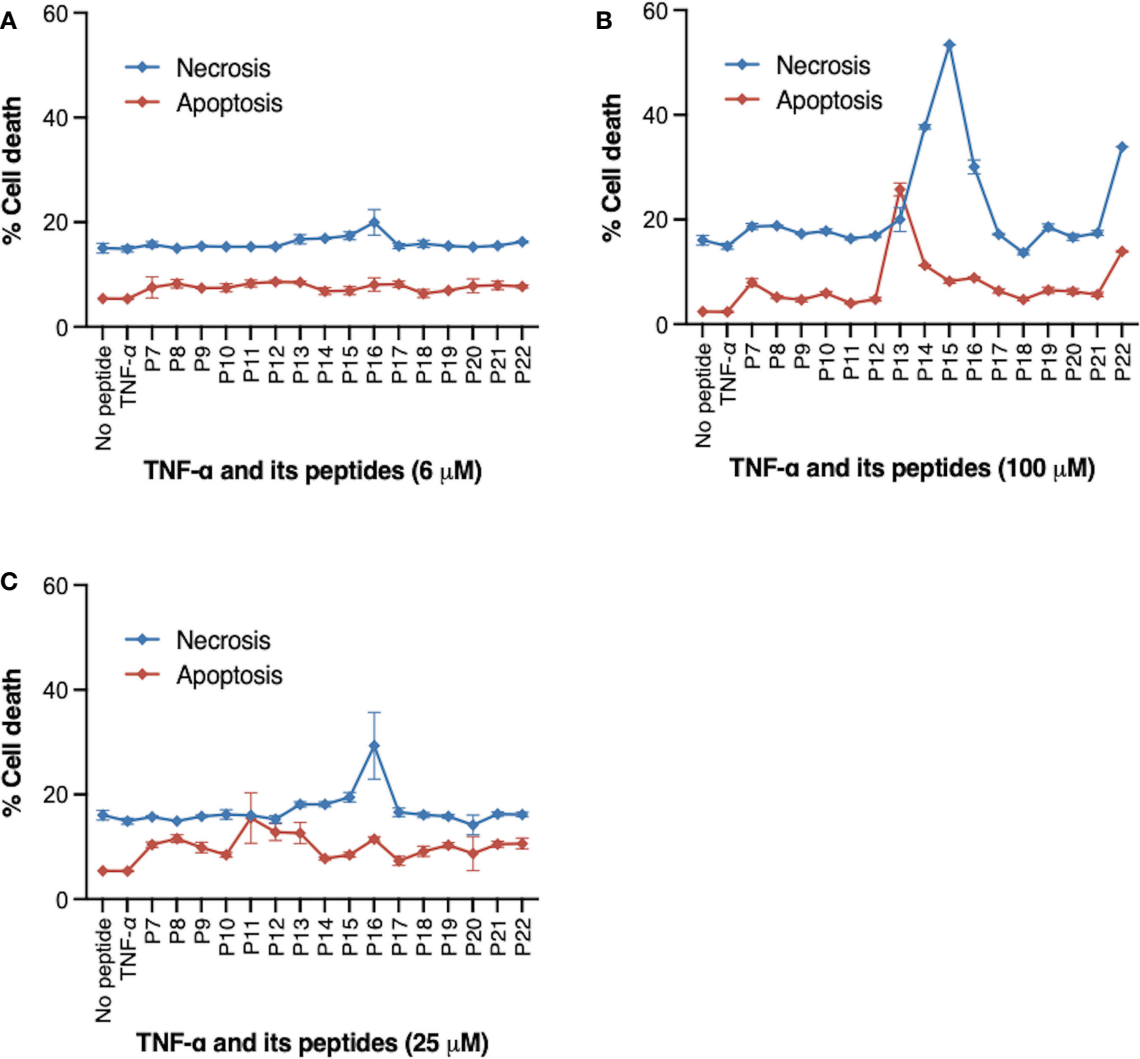
Peptides derived from TNF and their effect on cell death. MT2 cells were incubated with TNF protein (20 ng/ml), TNF peptides (P7-P22) at indicated concentrations, or DMSO (no peptide control) for 24 hours. **(A)** 6 μM **(B)** 50 μM **(C)** 100 μM. Frequency (%) of cell death was identified by Caspase-3 and live-dead dye double staining and measured by flow cytometry. P13 peptide at 100 μM induced high frequency of cell apoptosis (indicated by % of Caspase-3+ live-dead- cells). P14, P15, and P16 peptides at 100 μM induced predominantly cell necrosis (indicated by % of Caspase-3- live-dead+ cells). Data are from four independent experiments.

We have previously identified peptide fragments within TNF that are responsible for its pro-apoptotic and pro-necrotic functions (21). We first screened the TNF-derived peptides at different concentrations in MT2 cells for their effect on cell death in MT2 cells (**Figures 3A–C**). MT2 cells were the viral host cells in our *in vitro* HIV infection model. MT2 cells were incubated with either TNF protein, indicated TNF-derived peptides (P7-P22), or DMSO (no peptide control) for 24 hours. Induction of cell death was identified by caspase-3 and live-dead dye double staining and measured by flow cytometry. Differential peptide-induced cell death was found at concentration of 100 μM, but not so distinct at 6 or 25 μM. At the baseline level, ~5% of cell death from apoptosis and ~18% from necrosis. Addition of P13 at 100 μM induced a particular high frequency (28%) of apoptotic (Caspase-3^+^ live-dead^−^) (**Figure 3C**). P14, P15 and P16 at 100 μM induced high frequencies (39-56%) of necrotic (Caspase-3^−^ live-dead^+^), this was in agreement with our previous finding (24). Among three pro-necrosis peptides, P16 induced a similar degree of cell necrosis to that of cell apoptosis induced by pro-apoptosis peptide P13.

**Figure 4:**
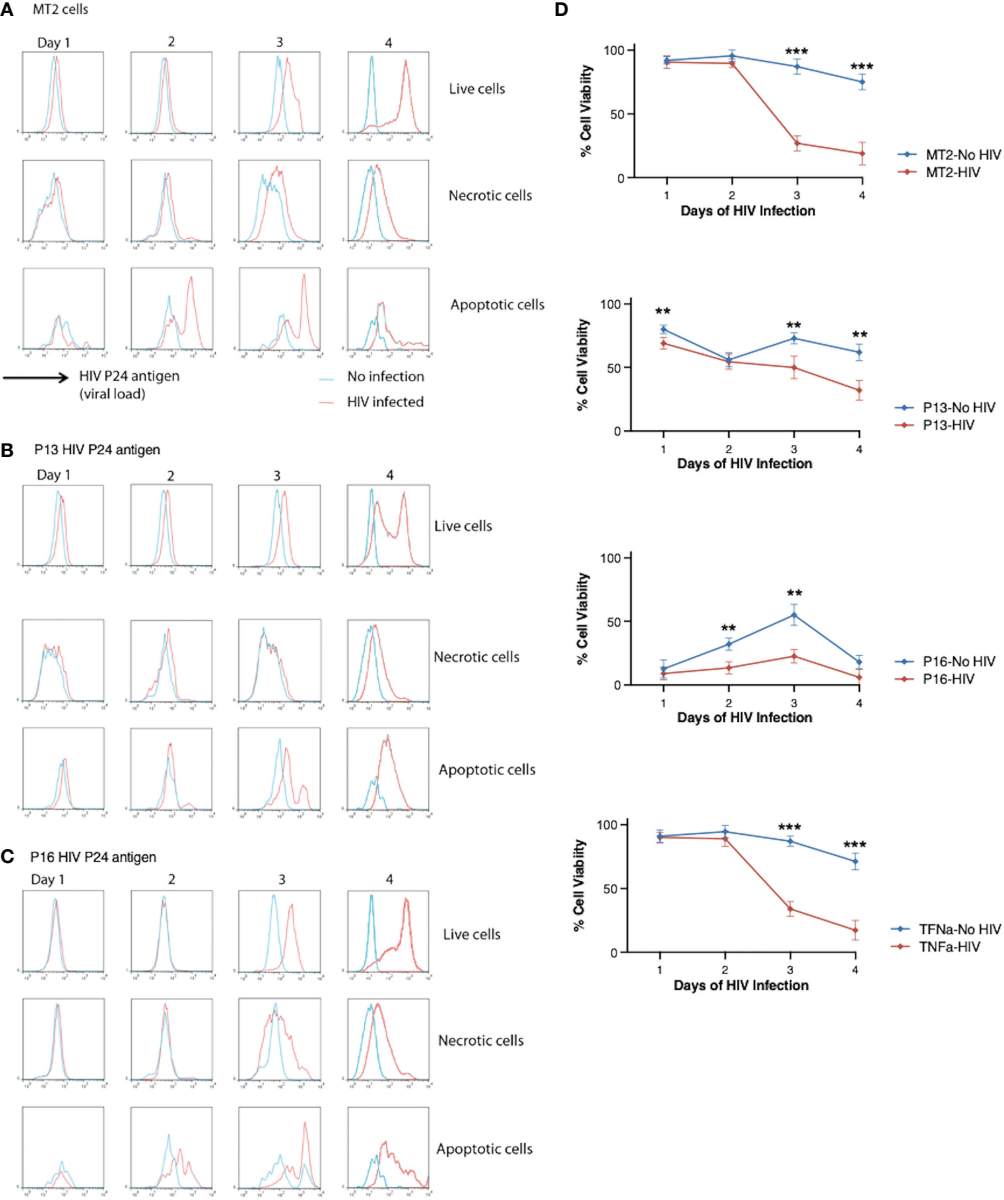
Effect of P13 and P16 on viral load and cell death in MT2 cells infected with HIV. **(A)** MT2 cells were infected at day 0 with HIV IIIB (MOI 0.001). Caspase-3 and live-dead dye staining were used to identify live (Caspase-3− live-dead dye-), necrotic (Caspase-3− live-dead dye+), and apoptotic (Caspase-3+ live/dead dye−) cells. HIV p24 stained with PE-conjugated anti-p24 antibody was used as a measure of viral load and measured by flow cytometry over 4 days. **(B)** MT2 cells were infected as before in presence of P13 peptide (100 μM). **(C)** MT2 cells were infected as before in presence of P16 peptide (100 μM). **(D)** Cell viability was measured in HIV-infected or non-infected MT2 cells in presence or absence of P13, P16 peptide (100 μM) or whole TNF protein (20 ng/ml). Red lines indicate p24 levels **(A-C)** or cell viability **(D)** in HIV-infected cells and blue lines indicate those in non-infected cells. Data are from four independent experiments. **p < 0.01, ***p < 0.001 (Multiple unpaired t-test).

We then asked whether induction of cell death in HIV-infected cells can constrain HIV spreading and/or prolong cell survival. We examined the effect of pro-apoptotic P13 and pro-necrotic P16 on viral load (indicated by p24 antigen levels) in HIV-infected MT2 cells compared with non-infected cells. Viral load increased in live, necrotic and apoptotic MT2 cells over 4 days of HIV infection (**Figure 4A**). Incubation of cells with pro-apoptotic peptide P13 slowed viral growth indicated by the divided p24 peaks in live cells at day 4 (**Figure 4B**, upper panel) in comparison to infected cells without exposure of the peptide (**Figure 4A**). Incubation of cells with pro-necrotic peptide P16 had little effect on viral growth (**Figure 4C**), displaying a high level of p24 detected in live cells similar to that of infected cells without exposure of the peptide (**Figure 4A**). We then examined whether peptide induction promotes overall cell survival. Cell viability (indicated by frequencies of Caspase-3^−^ live-dead^−^ cells) decreased significantly less rapidly in HIV-infected cells incubated with P13 over 4 days in comparison to infected cells without exposure of the peptide (**Figure 4D**). In the presence of P16, cell death occurred earlier, with some recovery at day 3, before decreasing again at day 4 in both HIV infected and non-infected cells (**Figure 4D**). TNF was found to have no effect on HIV induced cell death (**Figure 4D**). Together, these data suggest that pro-apoptotic peptide P13 has the potential to limit viral load and prolong survival of cells under infection.

## Discussion

HIV controls cell death to its advantage, producing the early and then chronic phases of infection (25). A cure for HIV, using stem-cell transplantation, has so far only been achieved in one patient with acute myeloid leukemia (26). The different mechanisms of cell death in HIV infection and the consequences of these on pathogenesis, for example viral transmission and reservoir formation, provide additional drug targets. Modulation of cell death pathways alongside effective HAART may provide a cure for HIV infection, by preventing loss of CD4^+^ T cells in early stages, and removing the viral reservoir in late stages (27).

We found in this study both apoptosis and necrosis in latent HIV infected patients and *in vivo* models of HIV infection (**Figures 1**, **2**). A bigger portion of cell death in HIV-infected patients is due to necrosis (**Figure 1**). In the *in vitro* infection experiment, induction of apoptosis, but not necrosis, with pro-apoptotic TNF peptide in HIV-infected cells slows down HIV-related cell death and reduces HIV viral load. Furthermore, pro-necrotic peptide has little effect on HIV-related cell death and viral transmission.

These results suggest a potentially novel therapeutic strategy for HIV infection – i.e. induction of apoptosis in HIV-infected cells. We hypothesize that induction of apoptosis in HIV-infected T-cells reduces the viral load (as shown in **Figure 4B**) because apoptosis leads to the death of infected cells before they have a chance to produce large numbers of viral particles. When a T-cell is infected with HIV, the virus uses the cell’s own machinery to replicate itself and spread to other T-cells. If the infected T-cell is not eliminated by the immune system or by induction of apoptosis, it can continue to produce new viral particles, leading to an increase in the viral load. When apoptosis is induced in infected T-cells, the cell dies in a controlled manner, preventing it from releasing large numbers of viral particles into the body. This effectively reduces the number of infected cells and thus the overall viral load. This has been proved by this study (**Figure 4B**). Additionally, apoptosis also prevents the spread of the virus to other T-cells, reducing the number of new infections.

We identified necrosis and apoptosis to be the major mechanisms of cell death in CD4^+^ lymphocytes in the latent HIV infection. We also found that double negative (CD4^-^/CD8^-^) lymphocytes, likely T, B, and natural killer (NK) cells, died by apoptosis and necrosis. As they are not targeted by the virus like CD4^+^ and CD8^+^ T cells, suggesting the presence of a bystander effect. One possible explanation for this bystander effect in HIV patients is that the virus can infect and kill CD4^+^ and CD8^+^ T cells, which can release signaling molecules, such as cytokines or chemokines, that can induce necrosis in neighboring cells, including double-negative lymphocytes. Additionally, the immune response mounted against the virus can also contribute to the destruction of neighboring cells by the release of cytotoxic molecules from the activated immune cells. The death of these double negative lymphocytes may contribute to the impairment of the immune system. Studies have also found that HIV infection can cause the loss of regulatory T (Treg) cells (24) and that a small percentage of Treg cells known to be CD4/CD8 double negative (28) might exhibit similar sensitivity to HIV infection like CD4^+^ Tregs. Resting double negative T cells were also found to express HIV viral proteins, driving CD4 receptor internalization, and potentially contributing to the viral reservoir (29). Furthermore, studies have shown that HIV infection can decrease B lymphocyte numbers, change NK cell homeostasis, and impair their antiviral effector functions (30, 31). Further research is needed to fully characterize the phenotype of the double negative cells identified in this study and to understand the mechanisms that lead to their death.

In this study, we sought to identify the major types of cell death in HIV infection and the effects of TNF peptides on latent infection. The finding of this study suggests that induction of apoptosis in HIV infected cells benefits in restriction of HIV spreading. Previously, models of HIV activation by inducing apoptosis have been tested in order to target the latent reservoir (32). Further work could explore the effects of peptides on cell death in the chronic phase of infection, in an attempt to reduce the viral reservoir.

## Data availability statement

The original contributions presented in the study are included in the article/supplementary material. Further inquiries can be directed to the corresponding authors.

## Ethics statement

The studies involving human participants were reviewed and approved by Ethics Committee, Beijing Youan Hospital, Capital University, Beijing, China. The patients/participants provided their written informed consent to participate in this study.

## Author contributions

QC performed the majority of experiments and data analysis. WL and C-HC conducted some of the experiments and analysis. YHZ, YZ and JZ collected and provided clinical samples. C-HC and SJ designed the project. QC, C-HC, and SJ wrote the manuscript. All authors contributed to the article and approved the submitted version.
